# *In vitro* pharmacodynamics of the antimalarial drug cabamiquine

**DOI:** 10.1128/aac.00802-26

**Published:** 2026-09-09

**Authors:** Claudia Demarta-Gatsi, Julia S. Wallner, Satish K. Dhingra, Sunil K. Narwal, Vandana Thathy, Mohamed Maiga, Alhassane B. Diallo, Maike Bonsen, Emilie Mathis, Ana Rita Gomes, Matthias Jacobs, Matthias Rottmann, Laurent Dembele, David A. Fidock, Julien Guizetti, Thomas Spangenberg

**Affiliations:** 1 Global Health R&D of Merck Healthcare, Ares Trading S.A. (a subsidiary of Merck KGaA, Darmstadt, Germany), Eysins, Switzerland; 2 Centre for Infectious Diseases, Heidelberg University Hospital 27178, Heidelberg, Germany; 3 Department of Microbiology & Immunology, Columbia University Irving Medical Center 21611, New York, New York, USA; 4 Center for Malaria Therapeutics and Antimicrobial Resistance, Division of Infectious Diseases, Department of Medicine, Columbia University Irving Medical Center 21611, New York, New York, USA; 5 Faculty of Pharmacy, Université des Sciences, des Techniques et des Technologies de Bamako (USTTB), Parasites and Microbes Research and Training Centre (PMRTC) 225803, Bamako, Mali; 6 LPHI, Université de Montpellier, CNRS, INSERM 27037, Montpellier, France; 7 Merck KGaA 2792 , Darmstadt, Germany; 8 Department of Medical Parasitology and Infection Biology, Swiss Tropical and Public Health Institute, Allschwil, Switzerland; 9 University of Basel, Basel, Switzerland; 10 Division of Infectious Diseases, Department of Medicine, Columbia University Irving Medical Center 21611, New York, New York, USA; Houston Methodist Hospital and Weill Cornell Medical College, Houston, Texas, USA

**Keywords:** *Plasmodium*, cabamiquine, pharmacodynamics, MitoTracker, live cell imaging, luminescence

## Abstract

Detailing the mode-of-action of novel antimalarial drugs is important to predict and optimize their treatment efficacy. To complement standardized *in vivo* studies, an *in vitro* pharmacodynamic characterization of the *Plasmodium* elongation factor 2 inhibitor cabamiquine has been undertaken on *Plasmodium falciparum*-infected red blood cells. Parasite growth arrest and killing kinetics were assessed using a MitoTracker assay, two genetically engineered luminescent parasite lines expressing firefly luciferase or NanoLuc (BRRoK assays), standard growth inhibition assays, and long-term live cell imaging. The results confirm that cabamiquine is a potent and fast-acting inhibitor of parasite protein translation, inducing complete arrest of the translational machinery within 6 h of drug exposure. Rate-of-kill assays showed a >100-fold difference in potency between the block on translation (luciferase-based BRRoK IC_50_ = 0.9 nM) and overall parasite killing (6 h-treatment, ring-stage standard growth inhibition assay IC_50_ > 100 nM), highlighting that translational arrest occurs well before parasite death. Prolonged inhibition of protein synthesis drives parasite exhaustion, followed by irreversible cell death within 48 h, preceding the parasite clearance observed in patients. These *in vitro* findings help contextualize why the clinically observed lag in parasite clearance may be temporally distinct from symptomatic improvement, including fever resolution. Here, cabamiquine presents itself as a fast-acting, slow clearing antimalarial drug.

## INTRODUCTION

Malaria is caused by intracellular parasites of the genus *Plasmodium* and remains a severe burden on global health (1). Recently, a resurgence in cases has been observed, resulting in over 282 million patients and approximately 610,000 malaria-related deaths in 2024 (2). One major challenge in combating the disease is the emergence of resistance to antimalarial drugs (3). A notable example is chloroquine, a hemozoin formation inhibitor, widely used from the 1940s until the 1960s, before widespread drug resistance emerged in Africa (4). More recently, treatment failures with frontline artemisinin-based combination therapies have highlighted the urgent need for novel drugs (5–7). Strengthening antimalarial drug development and prolonging regimen efficacy hinges on accurate assessment of both the rapidity and effectiveness of drug treatments.

**Fig 1:**
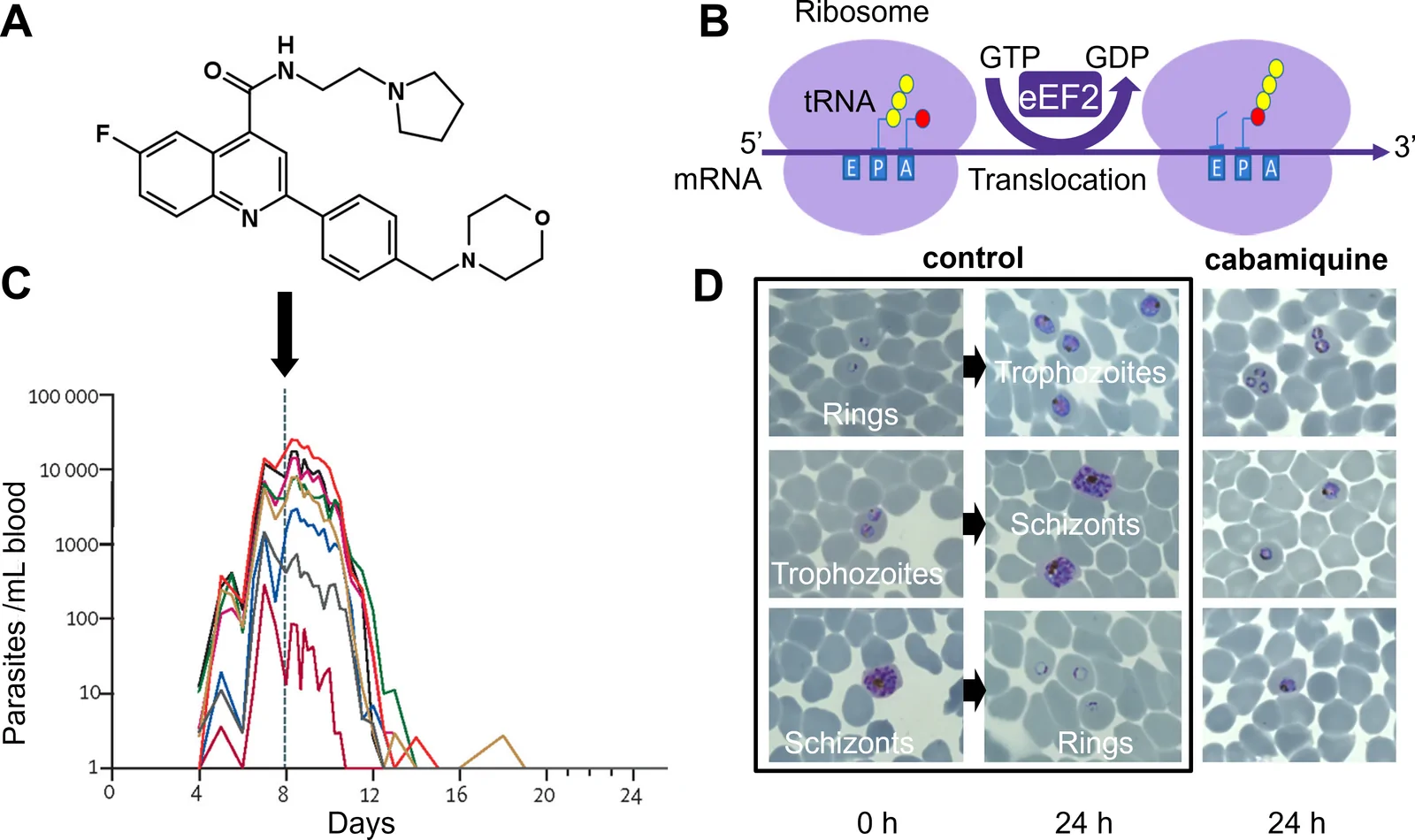
Overview of cabamiquine’s *in vitro* and *in vivo* profile. (**A**) Chemical structure of CBQ; (**B**) eEF2 protein function; (**C**) parasite clearance time volunteers infected with blood stage *P. falciparum* dosed with 800 mg of CBQ (succinate salt); (**D**) thin blood smear on synchronized rings, trophozoites, and schizonts untreated and treated with cabamiquine.

Identifying new drugs with novel modes-of-action (MoA) is, therefore, a priority in malaria drug discovery. One promising candidate is cabamiquine (CBQ, formerly known as M5717, DDD107498, Fig. 1A), which targets the *Plasmodium* eukaryotic translation elongation factor 2 (*Pf*eEF2, Fig. 1B) causing potent and selective inhibition of protein synthesis (8, 9) with blockade of the trophozoites when affecting blood stages of the parasite (Fig. 1D). CBQ’s long half-life (*t*_1/2_, ~150 h), multi-stage and -strain antiplasmodial activity (10), and high potency make it an attractive drug. CBQ has successfully completed Phase I safety trials along with a blood-stage volunteer infection study (11). During the trial, a delayed parasite clearance from the blood stream was observed and characterized as biphasic with an initial slow clearance, i.e., lag-phase (lasting 35–55 h), followed by rapid clearance (half-life 3.5 h to 6.4 h) (12) (Fig. 1C). In a recent Phase IIa clinical trial (NCT05689047), CBQ was combined with pyronaridine (PYRO) (13), which showed that all patients cleared their baseline parasitaemia with clearance times of 3 to 10 days (CBQ 330 mg:PYRO 360 mg; CBQ 660 mg:PYRO 720 mg) and no difference in clearance times between adults and adolescents. A median fever clearance time of 6.2 h (range: 6–17.9 h) was observed (14). Malaria signs and symptoms were present in all patients at screening, declined rapidly after dosing, and disappeared for all patients within 2 days then remaining zero through day 43.

These clinical observations highlight that the distinction between parasite killing and parasite clearance kinetics, which are usually thought to be linked, remains a challenge during the characterization of antiplasmodial pharmacodynamics (PD) (15). For CBQ, *in vitro* studies have seen a killing lag-phase (48 h) using classical parasite reduction rate (PRR) assays, which detect parasite resurgence after washout of the drug (8, 16). However, common metrics like parasite PRR and parasite clearance time (PCT) do not necessarily fully recapitulate the MoA of the drug (15), i.e., the discrimination between the action of the drug such as protein synthesis arrest, the ensuing parasite death (killing), and finally lysis of the infected red blood cell (iRBC). Additionally, metabolic tests, such as lactate dehydrogenase activity assays, cannot readily differentiate between cytostatic and cytotoxic effects.

Here, we endeavored to discriminate these various PD elements of CBQ *in vitro*. We employed different strategies using (i) specific dyes, i.e., MitoTracker (MT); (ii) bioluminescence relative rate-of-kill (BRRoK) assays using two types of genetically engineered luminescent parasites compared to standard growth inhibition assays; and (iii) live cell imaging of *Plasmodium*-infected red blood cells (iRBCs) to better characterize the MoA in terms of parasite growth arrest and killing kinetics.

## RESULTS

### CBQ compromises parasite mitochondrial integrity within 12 h of exposure

**Fig 2:**
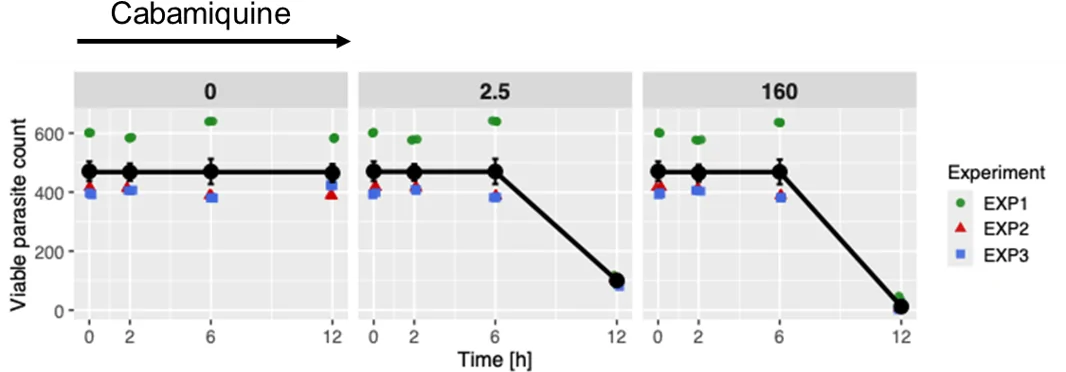
Effect of cabamiquine on parasite viability over 12 h, assessed by mitochondrial activity with MitoTracker (MT) staining. Parasite viability was measured at 0, 2.5, 6, and 12 h after cabamiquine exposure. Model predictions from the final model (lines) and original data (points) from three biological replicates (EXP1, EXP2, and EXP3). The numbers in each panel represent the concentration of cabamiquine in nM in each well. Data are presented as viable parasite counts over time, referring to the total SYBR Green and MT positive parasite count measured by flow cytometry.

To assess the immediate impact of CBQ on the viability of asexual blood stages, asynchronous *P. falciparum* 3D7 parasites were exposed *ex vivo* to CBQ, and mitochondrial activity was monitored with MT as a live-cell viability probe (17). CBQ-treated parasites showed a clear and reproducible decline in viable parasite counts, defined as MitoTracker (MT) and SYBR Green-positive parasites, beginning 6 h after drug exposure and progressing through 12 h. In contrast, untreated control cultures retained high viable parasite counts and continued to grow over the same interval (Fig. 2). These findings suggest that inhibition of eEF2 disrupts metabolic processes that compromise mitochondrial membrane potential, as reflected by the diminished MT positive parasites within 12 h.

### CBQ rapidly arrests protein translation and requires prolonged inhibition to achieve full parasiticidal activity

To validate these observations, we employed three complementary assays: (i) a bioluminescence relative rate-of-kill (BRRoK [18]) assay using a Dd2 line expressing firefly luciferase (Dd2^luc^) under the control of the *Pfhrp3* promoter, which served as a surrogate marker for PfeEF2 activity; (ii) a second BRRoK assay using a Dd2 line expressing NanoLuc (Dd2^KnL^) driven by the *Pfcalmodulin* promoter, which served as an independent bioluminescence readout; and (iii) a standard 48 h growth inhibition assay. We used two BRRoK reporter lines because their driving promoters differ in stage-specific activity relative to *PfeEF2* expression: the *Pfhrp3* promoter driving luciferase is active in rings and early trophozoites, closely tracking PfeEF2, whereas the *Pfcalmodulin* promoter driving NanoLuc is less tightly regulated and is more highly expressed in trophozoites and schizonts, providing an independent, complementary readout. CBQ’s effects were compared between synchronized ring (0–6 h post-invasion, hpi) and trophozoite (24–32 hpi) stages to assess possible differential killing kinetics across blood stages, with exposures of 6, 24, or 48 h.

**Fig 3:**
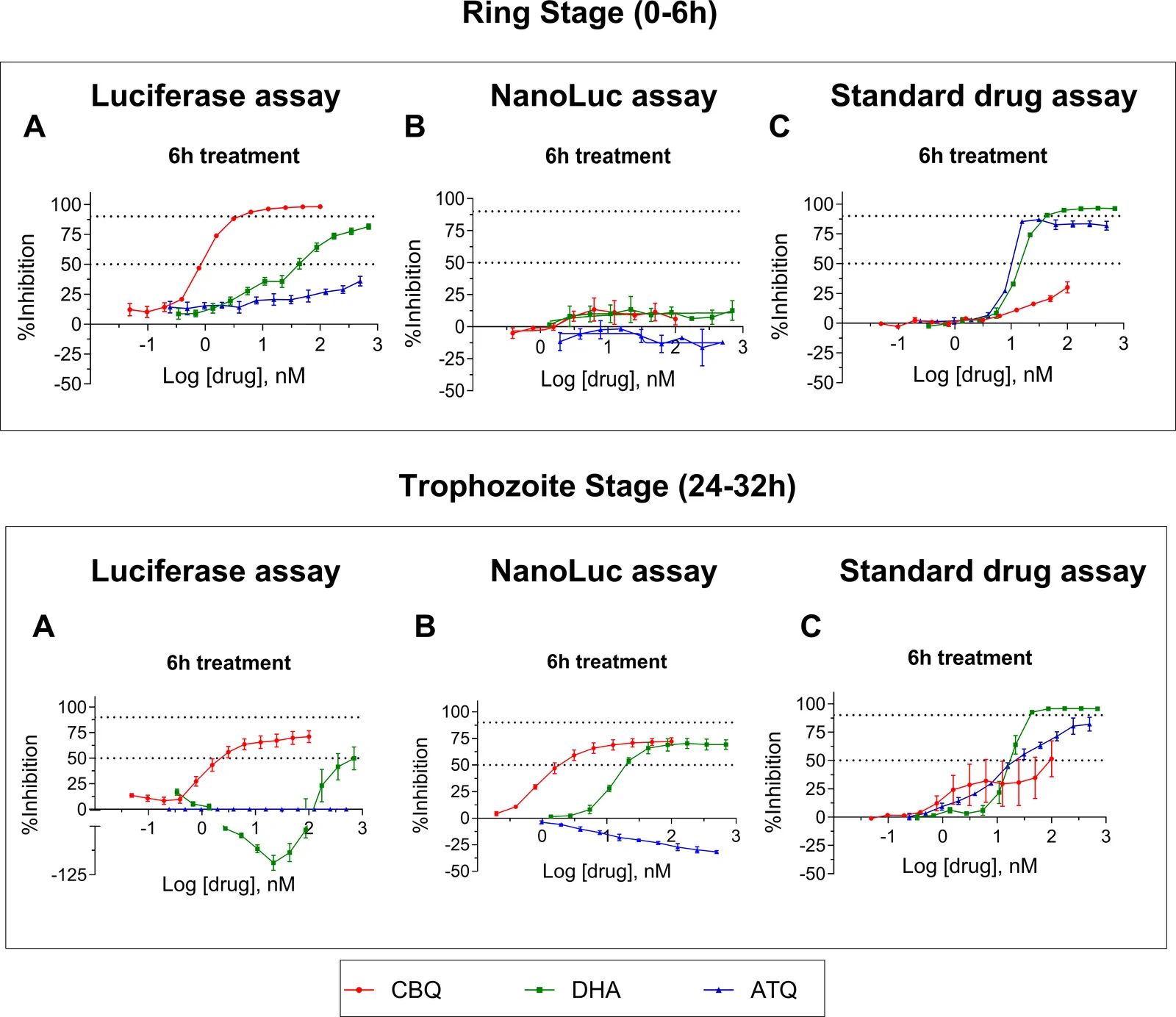
Comparative dose-response profiles of ring or trophozoite stages exposed to 6 h pulses of cabamiquine (CBQ), dihydroartemisinin (DHA), or atovaquone (ATQ). Synchronized ring (0–6 h post-invasion) or trophozoite (24–32 h post-invasion) stages were treated with CBQ, DHA, or ATQ for 6 h, and drugs were washed off and plates were incubated until the 48 h endpoint. Data were generated from three distinct assays. (**A**) Firefly luciferase activity in Dd2^luc^ parasites. (**B**) NanoLuc activity in Dd2^KnL^ parasites. (**C**) Standard growth inhibition assay assessed by flow cytometry (SYBR Green and MT dual labeling) using the Dd2^luc^ line. Data represent means ± SEM from 2 to 5 independent assays, each performed in duplicate. Dotted lines indicate 50% and 90% inhibition thresholds.

Using Dd2^luc^, a 6 h pulse of CBQ rapidly inhibited protein translation in both rings and trophozoites, with an IC_50_ of 0.9 nM (rings) and 2.9 nM (trophozoites), far lower than dihydroartemisinin (DHA) (6 h IC_50_ ≈ 46.8 nM in rings, *P* = 0.077; ≈166.2 nM in trophozoites, *P* = 0.223), indicating CBQ’s fast action on PfeEF2 (Fig. 3A; Table S1). This rapid translational inhibition by CBQ was corroborated in NanoLuc-expressing Dd2^KnL^ trophozoites, where a similarly potent and fast signal was observed with a 6 h IC_50_ of 2 nM (vs 20.4 nM for DHA, *P* = 0.0085), which was sustained at 24 h (CBQ IC_50_ 5.1 nM, vs 41.0 nM for DHA, *P* = 0.0407); however with this nanoLuc line CBQ produced a 6h IC_50_ >100 nM (Fig. 3B; Table S2). This discrepancy between IC_50_ values between Dd2^luc^ and Dd2^KnL^ rings may be a result of the *Pfcalmodulin* promoter’s higher activity in trophozoites. At the ring stage, the NanoLuc assay’s lower basal expression, together with the longer intracellular half-life of NanoLuc protein (>6 h vs ~2 h for firefly luciferase), may have prevented the detection of translational inhibition after only a 6 h pulse. Nonetheless, NanoLuc ring-stage parasites exposed to CBQ yielded IC_50_ values of 0.8 nM after 24 h (compared with 5.9 nM for DHA, *P* = 0.299) and 3.0 nM after 48 h (compared with 23.6 nM for DHA, *P* = 0.163). These data confirm that the NanoLuc reporter remains functional in rings given more time (Table S2). In contrast, the *Pfhrp3*-driven luciferase assay reliably detected inhibition across both stages and all pulse durations tested, reaching significance against both DHA and atovaquone (ATQ) at the trophozoite 24 h timepoint (*P* = 0.0010 for each) and against DHA at 48 h (*P* = 0.0070) (Fig. S1 and S2; Table S1).

In the standard growth inhibition drug assay, using the same 6 h CBQ exposure and 48 h recovery period, CBQ inhibited only ~30% of parasite growth in rings (IC_50_ > 100 nM), indicating slower parasite killing than was suggested by the luciferase-based BRRoK assay, which directly reported the rapid loss of firefly luciferase activity following the inhibition of protein translation. DHA inhibition was more potent (6 h IC_50_ = 15.4 nM; Fig. 3; Table S3). Trophozoites were more sensitive to CBQ at this timepoint (6 h IC_50_ = 17.6 nM) though still less potent than the luciferase-assay 6 h IC_50_ measurement of 2.9 nM. Extending exposure to 24 h or 48 h markedly improved growth inhibition, with CBQ IC_50_ values of 3 nM and 1.2 nM, respectively, when starting with rings, or ~1 nM for both exposures when starting with trophozoites (Table S3). Of note, the negative inhibition observed at low concentrations of DHA in the luciferase and NanoLuc assays likely reflects parasite recrudescence following sub-lethal DHA exposure, an artifact of the bioluminescence readout rather than true growth stimulation. DHA remained faster and more complete in killing across stages at shorter durations, while ATQ showed stage-specific effects (Fig. S1 and S2; Tables S1 to S3).

Overall, CBQ rapidly blocked protein synthesis and induced immediate translation arrest, but its parasite-killing kinetics were slower in the short term (6 h), particularly at the ring stage, and became near-complete by 48 h across both stages. These findings were consistently supported across both BRRoK reporter lines at the trophozoite stage, with divergence between them at the ring stage attributable to differences in promoter activity relative to *PfeEF2* expression, not to CBQ’s MoA or to the smaller number of replicate experiments performed for the NanoLuc assay (*N* = 2 vs *N* = 3–6 for luciferase).

### Real-time live-cell imaging reveals rapid CBQ-induced parasite growth arrest followed by death at single-cell resolution

**Fig 4:**
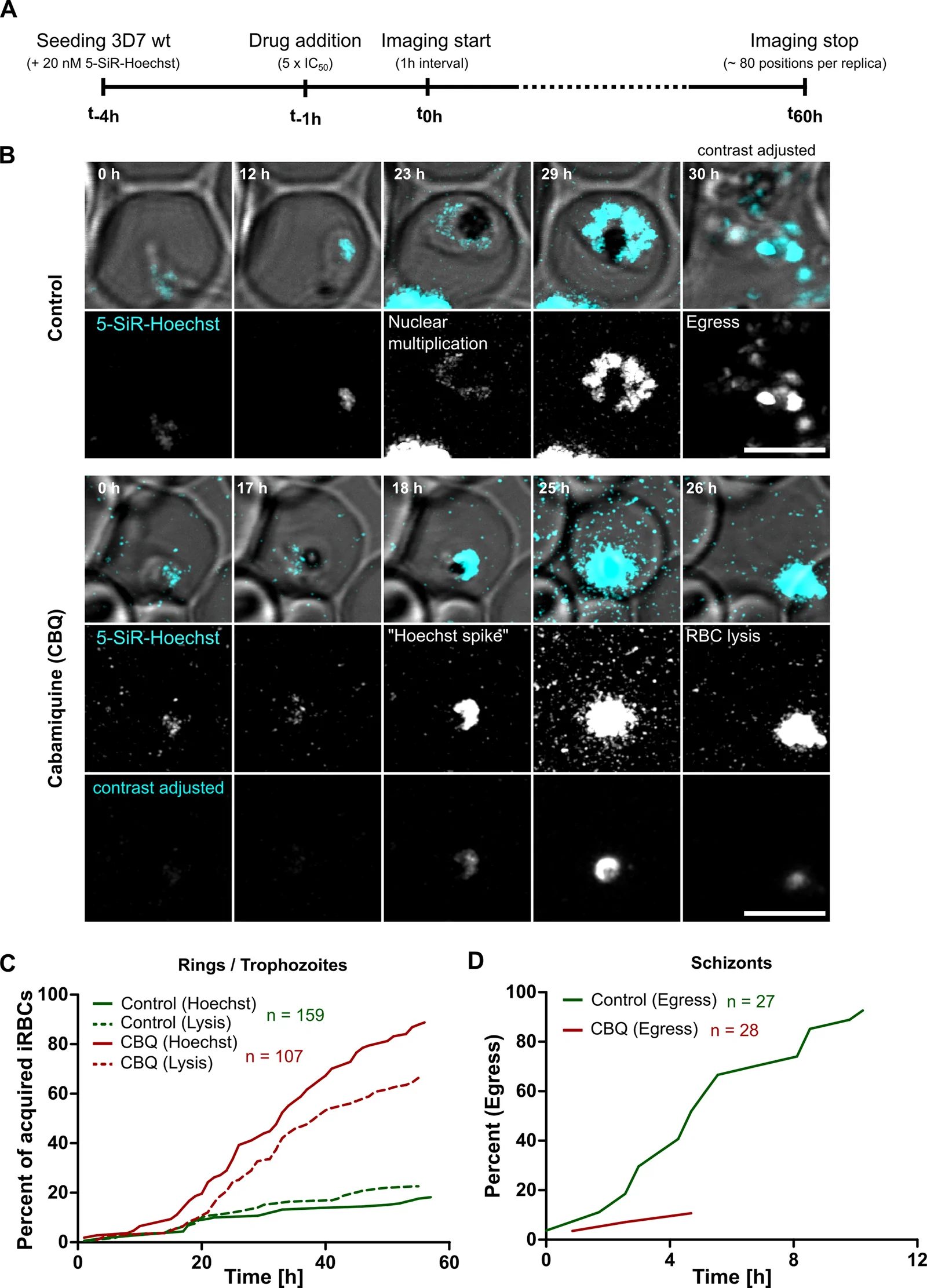
Long-term time-lapse microscopy of death-associated events in drug-treated *P. falciparum*-infected red blood cells (iRBCs). (**A**) Schematic timeline of parasite seeding into imaging dishes, labeling, treatment conditions, and imaging protocol. (**B**) Representative stills from time-lapse movies acquired of 3D7 parasites labeled with 20 nM 5-SiR-Hoechst (cyan) on Zeiss LSM 900 Airyscan that were either untreated or to which 5 nM CBQ were added. Transmission channel (gray). Due to the very strong fluctuation of the signal during a Hoechst spike and merozoite egress, the contrast-adjusted channels have been provided for adequate visibility. Scale bars, 5 µm. (**C**) Cumulative histogram of “Hoechst spike” and iRBC lysis events in control and drug-treated blood stage parasites over time. Data were pooled from three independent replicas for each condition. (**D**) Cumulative histogram of egress events in treated and untreated schizont stage parasites over time. Data were pooled from two independent replicas for each condition.

To assess the impact of 5 nM CBQ on individual *P. falciparum* blood stage parasites, we acquired time-lapse imaging data of the 3D7 wild-type strain using an Airyscan confocal microscope in low phototoxicity mode under physiological incubation conditions. To visualize parasite nuclei in addition to the iRBC morphology, we added 5-SiR-Hoechst, a next-generation infrared fluorogenic live cell dye, about 4 h before the start of the recording (Fig. 4A) (19–21). A sudden and drastic increase of 5-SiR-Hoechst fluorescence intensity prior to intraerythrocytic development cycle (IDC) completion can be interpreted as a loss of metabolic activity. The lack of growth after 5-SiR-Hoechst influx, the association with host cell lysis, and the substantially higher occurrence in drug-treated parasites suggest that it can be used as a proxy for parasite death or at least cytotoxic activity. Premature lysis of the iRBC can be considered a definitive parasite death marker in this obligate intracellular life cycle stage (Fig. 4B).

Drug or control was added to the imaging dish about 1 h before the start of image acquisition. Parasites of different stages were randomly selected from an asynchronous population, omitting schizont stages that had already begun dividing their nuclei. Parasites were then imaged for 60 h at 1 h time intervals. Most control parasites underwent a normal IDC (Fig. 4B). CBQ-treated parasites, however, showed a substantial and rapid increase in Hoechst fluorescence intensity. When detecting a >4-fold increase of maximum Hoechst signal intensity within 1–2 h, we designated it as a “Hoechst spike.” This spike was commonly followed by RBC lysis, marking parasite death. Furthermore, we could not detect substantial parasite nor hemozoin growth under drug treatment.

### CBQ abolishes parasite egress and nuclear multiplication with a 15-h lag phase preceding parasite death

Since none of the CBQ-treated parasites displayed a normal IDC progression, we quantified the occurrence of “Hoechst spikes” and iRBC lysis (Fig. 4C). Within 60 h, a Hoechst spike was observed in 18% of control cells, while drug treatment caused a statistically significant increase to 89% (*P* < 0.0001, Student’s *t*-test). The Hoechst spike was usually followed by iRBC lysis in 23% of cases for control untreated iRBCs, which increased to 69% for CBQ-treated iRBCs (*P* < 0.002, Student’s *t*-test) within the first 60 h. CBQ displayed a lag-phase of about 15 h before the onset of parasite death. Intriguingly, compared to about 80% of control parasites, none of the CBQ-treated ring and trophozoite stage parasites ever achieved egress or showed signs of nuclear multiplication, even though many of them should have done so within the acquisition time (Fig. S4).

### CBQ rapidly blocks normal IDC progression

These data raised the critical question of whether despite delayed killing the progression through the IDC was halted immediately through a rapid cytostatic effect of CBQ. To test this, we treated schizont-stage parasites that were approaching the end of their IDC with multiple nuclei, and imaged them as above for up to 18 h. A rapidly acting drug would likely prevent egress of these late-stage parasites, while control parasites would complete their cycle within this time. Indeed, 93% of control schizont parasites egressed within 11 h, as expected for a normal cell cycle progression (Fig. 4D). Of the CBQ-treated parasites, however, only 11% achieved egress. These events occurred only within 6 h after treatment and in schizonts that were already very close to the end of their cycle, suggesting a rapid cytostatic effect of the drug.

### Rapid intracellular accumulation of a fluorescent CBQ analog suggests fast drug uptake across all intraerythrocytic stages

**Fig 5:**
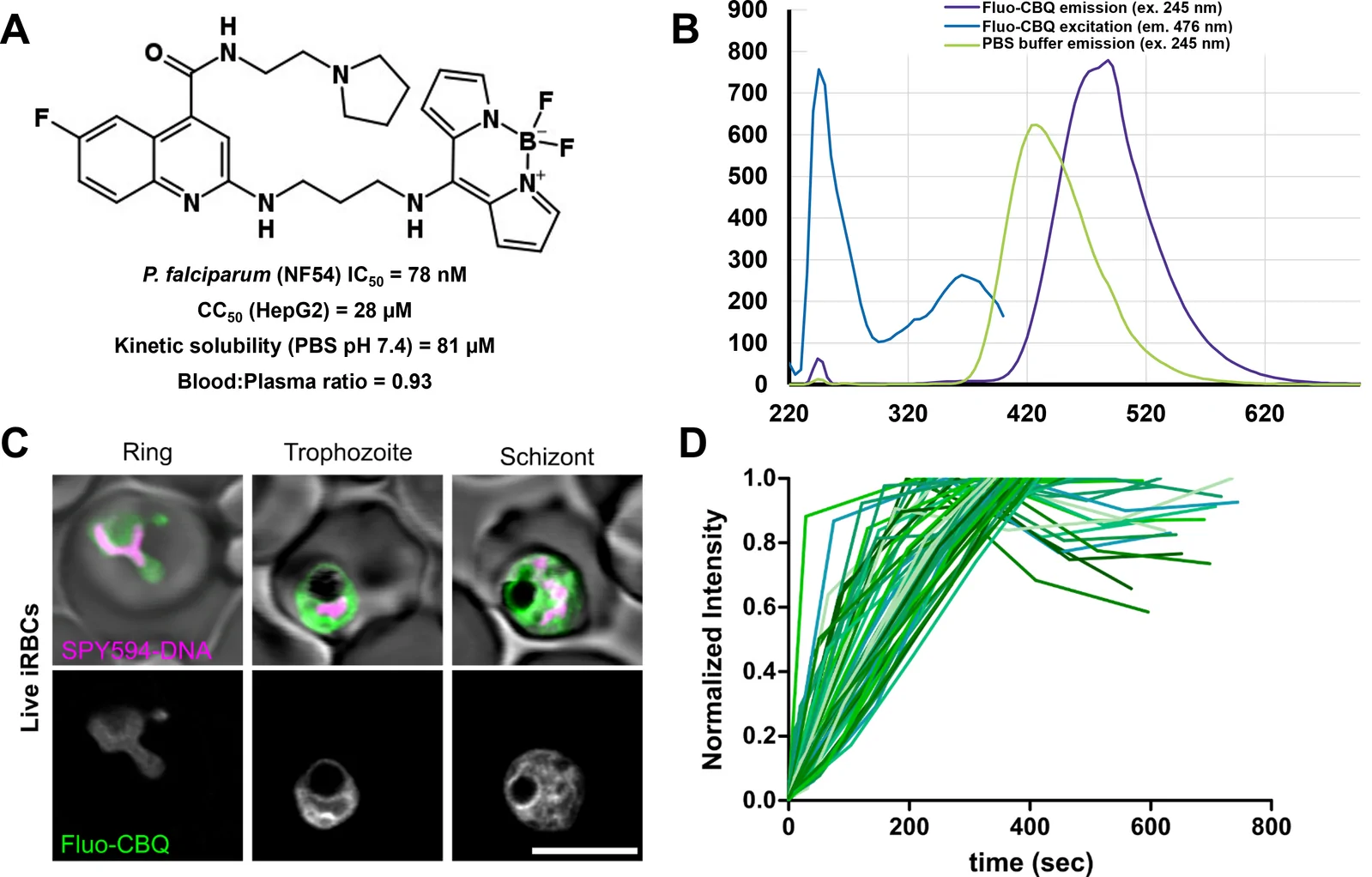
Accumulation of the fluorescent CBQ derivative in blood stage *P. falciparum*. (**A**) Molecular structure of Fluo-CBQ with key drug metrics; (**B**) fluorescence excitation and emission spectra of Fluo-CBQ exhibiting an emission maximum at 476 nm and an excitation maximum at 245 nm, with a secondary peak at 365 nm with approximately 1/3 of the intensity. The PBS control is shown with some fluorescence at 245 nm excitation; (**C**) LSM900 Airyscan confocal images of live parasites at different IDC stages labeled with 200 nM SPY594-DNA (magenta) and about 10 min after the addition of 100 nM Fluo-CBQ (green). The merge with transmission light channel is shown on top. Scale bar, 5 µm; (**D**) time curves of normalized Fluo-CBQ fluorescence intensities in parasites. 50 nM Fluo-CBQ addition occurred just after timepoint zero, and thereafter, images were taken at about 3.5 min intervals. Each line represents an individual cell (*n* = 56). Measurements were carried out in four technical replicates and two biological replicates.

Lastly, we wanted to assess via imaging whether CBQ uptake by the parasite was limiting for its *in vitro* activity. Previous work with our homology model suggested that key interacting amino acids with CBQ are tyrosine Y186, which was predicted to interact through π–π stacking with the quinoline, and the glutamate E134 that was predicted to interact with the charged pyrrolidine ring through an H-bond and ionic bonds (9). Hence, the exit vector from the quinoline moiety could be amenable to modification to include a fluorescent moiety (22). Here, we considered integrating a BODIPY (boron–dipyrromethene) dye as a fluorescent group and synthesized a derivative termed Fluo-CBQ (Fig. 5A, Materials and Methods). We confirmed that the compound was able to emit fluorescence with a wavelength of 476 nm with an excitation peak at 254 nm and a secondary one at 365 nm (Fig. 5B). Next, we assessed the growth inhibition activity using a hypoxanthine incorporation assay to avoid interference with the readout. We found that the *P. falciparum* asexual blood stage growth inhibition IC_50_ was 78 nM (NF54 strain*,* 72 h, Supporting information) for Fluo-CBQ.

Imaging DNA-labeled live iRBCs within 10 min of the addition of 100 nM of Fluo-CBQ showed a specific accumulation of fluorescent signal within the parasite, while the RBC remained unlabeled (Fig. 5C). The labeling occurred throughout the IDC but seemed to intensify in later stages. In trophozoites and schizonts, there was a more notable association with internal membranous structures. The lack of RBC membrane labeling and a weak parasite plasma membrane labeling suggest that this localization does not result from non-specific membrane binding. One possible explanation for the observed staining pattern, which aligns with the target of CBQ, is the enrichment at the ribosomes on the surface of the rough endoplasmic reticulum.

To quantify accumulation kinetics, we measured Fluo-CBQ signal in live parasites over time. After acquiring untreated parasites at *t*_0_, we added 50 nM Fluo-CBQ, started a time-lapse, and measured fluorescence intensity for up to 15 min (Fig. 5D). We noted a very rapid increase of fluorescence in all parasites, which reached maximal intensity within 10 min, suggesting that drug uptake kinetics are not limiting for antiparasitic activity.

## DISCUSSION

Blood-stage parasitemia is used as a biomarker in malaria patients, and parasite clearance time represents one of several parameters employed to assess the efficacy of antimalarial drugs. A long-standing assumption has been that fast-acting drugs lead to rapid removal of circulating parasites, whereas slow-acting drugs result in prolonged clearance time. Although this hypothesis has guided our interpretation of parasite clearance kinetics, it has been demonstrated that measuring the rate of decline in parasitemia after treatment reflects the rate of host removal of dead parasites rather than the rate at which the drug kills parasites (23). Indeed, the *in vivo* parasite clearance curve may not always reflect parasite viability, as dead or exhausted parasites can persist in circulation for extended periods prior to splenic elimination (15, 24).

CBQ, a protein synthesis inhibitor, acts by preventing ribosome translocation along mRNA through PfeEF2 inhibition, a mechanism fundamentally distinct from conventional antimalarials, as it paralyzes the protein synthesis machinery and initiates a molecular cascade that culminates in parasite death (25). Initial *in vitro* assessment using the PRR test system that measures parasite viability after serial dilutions and drug washout (16) suggested a slow killing profile with a 24–48 h lag phase (8). While this approach provides insight into immediate lethality, it cannot assess cidal activity after prolonged drug exposure, a limitation that may have misled the community as *in vitro* parasite clearance seems, at first glance, to replicate the *in vivo* outcome.

This ambiguity was further highlighted in Phase IIa clinical trials, where a lag-phase in parasite clearance from the bloodstream was observed when CBQ was co-administered with PYRO, despite fever resolution occurring by approximately 6 h post-treatment (15.5 h in Pyramax) (14), suggesting a dissociation between clinical symptoms and parasite clearance kinetics. However, in clinical settings, routine blood smear analysis does not distinguish viable from nonviable circulating parasites, fundamentally limiting the interpretation of killing kinetics *in vivo* (17).

**Fig 6:**
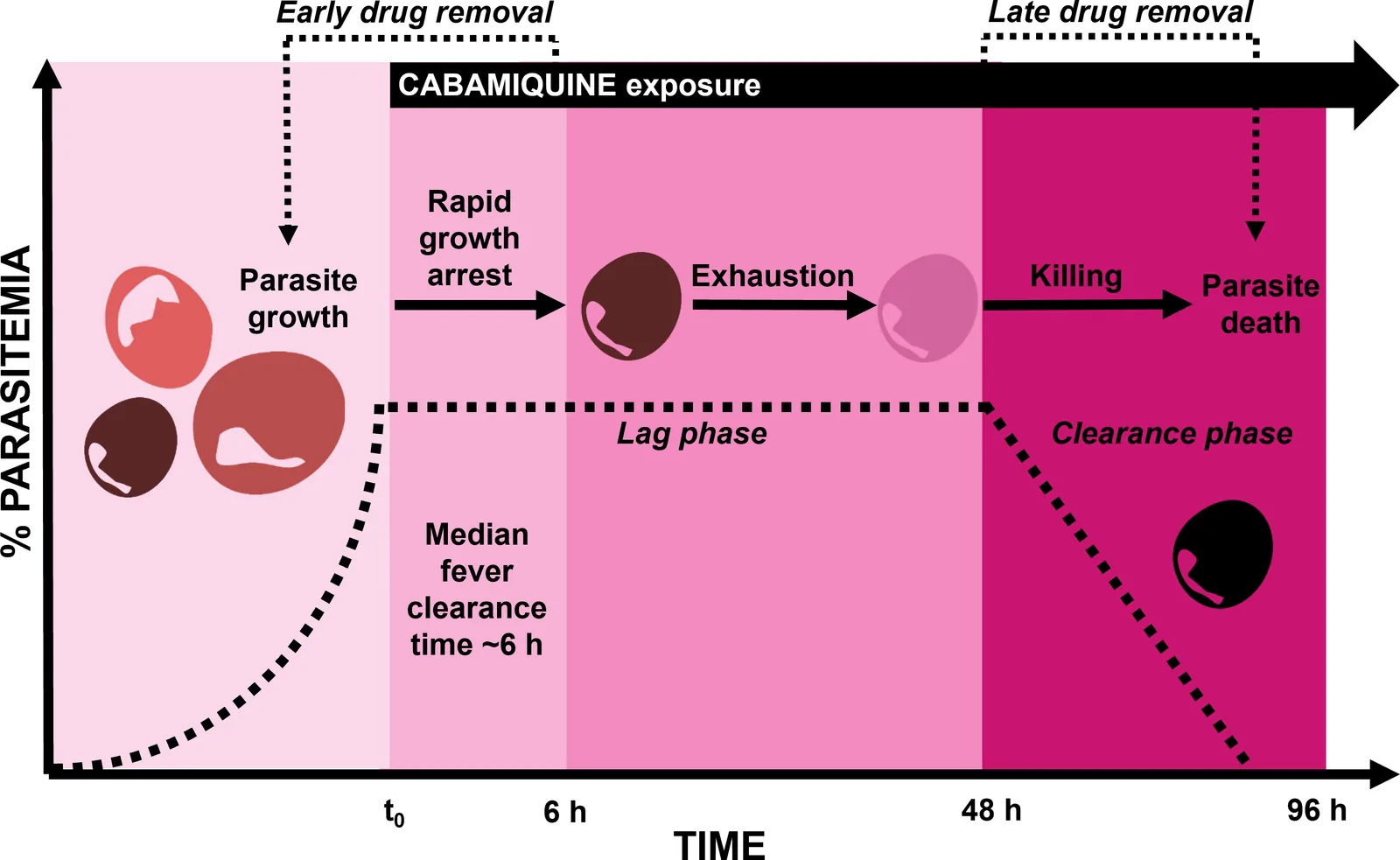
Schematic view of CBQ pharmacodynamic profile.

To determine whether the clinically observed lag in parasite clearance reflects true slow killing, cytostatic effects, or an alternative mechanism, we employed four complementary approaches: MT staining, luminescent BRRoK reporter parasites, a standard growth inhibition assay, and live-cell imaging of *Plasmodium*-iRBCs, to characterize CBQ’s PD behavior in terms of parasite growth arrest and killing kinetics. Using two BRRoK reporter lines, firefly luciferase (Dd2^luc^, *Pfhrp3* promoter) and NanoLuc (Dd2^KnL^, *Pfcalmodulin* promoter), CBQ produced complete arrest of the translational machinery within a 6 h pulse, with the luciferase assay detecting this rapid effect at both ring and trophozoite stages and the NanoLuc assay corroborating it at the trophozoite stage, where *Pfcalmodulin* promoter activity is highest; NanoLuc’s lower sensitivity in rings at this early timepoint reflects its promoter’s weaker activity during this stage rather than any difference in CBQ’s mechanism. In parallel, a standard growth inhibition assay using the same 6 h pulse showed CBQ inhibiting only a fraction of parasite growth by the 48 h endpoint, a substantially slower apparent effect than the BRRoK assays suggested. This divergence is not a contradiction but reflects what each assay measures: the BRRoK assays report CBQ’s direct, rapid effect on translation, whereas the standard assay reports the parasite’s eventual fate, which lags behind that initial translational block. The collected data indicate that CBQ is rapidly arresting the parasite protein synthesis machinery and that blockade of this machinery for a prolonged time in the presence of the drug leads to irreversible parasite damage, termed exhaustion, ultimately resulting in parasite death (Fig. 6). However, an early washout (e.g., 6 h) may permit parasite recovery, consistent with incomplete growth inhibition observed at this timepoint. Real-time imaging demonstrates that egress block, a functional surrogate for protein synthesis arrest, occurs within 6 h, consistent with rapid cytosol drug uptake. This is complemented by MT staining on *P. falciparum* 3D7 parasites where viability was lost from 6 to 12 h post exposure, supporting a rapid onset of cytostatic effects followed by a delayed but inevitable loss of viability.

These findings are corroborated by the structural and cellular data of Anton et al. (25), demonstrating that CBQ-treated parasites remain arrested in size and organellar content, in striking contrast to control parasites that grow dramatically over the same period. Following prolonged drug exposure (18 h post-treatment), an unusual nuclear accumulation of ribosome-like particles and enrichment of ribosome biogenesis and rRNA maturation pathways were observed, suggestive of a futile compensatory response to sustained translational stress and consistent with the progressive and irreversible parasite exhaustion described herein.

Additionally, the long half-life of cabamiquine ensures prolonged target engagement *in vivo*, whereby an artificial washout at early timepoints, as applied in the PRR assay, would permit parasite recovery from a PK/PD standpoint and therefore, underestimates, the true cidal activity of the drug in a clinical setting. *P. falciparum* asexual blood stages multiply within RBCs, which are cleared from the bloodstream by the spleen primarily when their deformability is altered. Based on its MoA, the slow clearance of circulating parasites observed clinically can be attributed to the inability to modify their RBC hosts (26), which is consistent with rapid arrest of protein synthesis.

From a broader clinical perspective, as observed on a small cohort of patients, clearance of symptoms such as fever within hours following drug administration can be achieved with an actual slow parasite clearance profile (14), underscoring the dissociation between symptomatic relief and parasitemia decline. This suggests that rapid control of parasite growth or impaired replication may translate into meaningful early clinical gains and that antimalarials with this cytostatic-to-cidal PD profile may represent a clinically relevant alternative to the fast-acting class of artemisinins that are achieving parasite clearance within hours. However, rapid parasite clearance may cause possible complications as massive numbers of parasites are destroyed within hours, potentially releasing enormous quantities of parasite-derived antigens and endotoxins into the bloodstream and triggering paradoxical exacerbation of fever, systemic inflammation, and clinical manifestations despite effective parasite elimination (27). In this context, a slower clearance profile as seen with CBQ may confer an advantage by avoiding this inflammatory burden.

Beyond direct killing, CBQ’s ability to induce parasite exhaustion, paralyzing the parasite without causing immediate lysis, may provide the host immune system with the time required to mount and sustain an effective anti-parasite response. For example, controlled blood-stage infection-and-treatment approaches, including immunization with genetically attenuated blood-stage parasites (28–30) or low-dose infection-and-treatment regimens (31, 32), exploit the erythrocytic stage itself to induce protective immunity, primarily through parasite-specific antibodies and CD4^+^ T-cell responses. Also, this immune-cooperative dynamic is well-established in other therapeutic areas that utilize cytostatic drugs, where drug-induced growth arrest precedes and enhances host immunity, ultimately leading to the elimination of pathogens or tumor cells (33, 34).

Finally, augmenting clinical monitoring with approaches capable of discriminating viable from nonviable circulating parasites after treatment (16) would substantially improve the interpretability of clearance kinetics and the assessment of drug efficacy in future trials.

### Conclusion

By employing four complementary experimental approaches, MT staining, genetically engineered luminescent parasites including both the firefly luciferase (Dd2^luc^) and NanoLuc (Dd2^KnL^) reporter lines, a standard growth inhibition assay, and live-cell imaging of *Plasmodium*-iRBCs, we have substantially advanced the mechanistic characterization of CBQ’s PD profile. CBQ is a potent and fast-acting inhibitor of parasite protein translation, inducing complete arrest of the translational machinery within 6 h of drug exposure, as evidenced by rapid bioluminescence loss in the luciferase and NanoLuc BRRoK assays at the trophozoite stage. At the ring stage, only the luciferase assay detected this fast arrest at 6 h; the NanoLuc assay required longer exposure (24–48 h), reflecting the *Pfcalmodulin* promoter’s lower and less tightly regulated ring-stage activity relative to *Pfhrp3*, rather than a difference in CBQ’s mechanism. Standard growth inhibition assays confirmed that this rapid translational arrest precedes complete parasite killing, which becomes near-complete only by 48 h, directly demonstrating that CBQ’s effect on its target is faster than its lethal effect on the parasite. Prolonged inhibition of protein synthesis drives parasite exhaustion, followed by irreversible cell death within 48 h, preceding the parasite clearance observed in patients. The clinically observed lag in clearance is, thus, attributable to the inability of translation-arrested parasites to continue remodeling their host erythrocyte, rather than delayed killing. Importantly, relief of clinical symptoms such as fever is temporally decoupled from the actual clearance mechanism. These insights open new opportunities in malaria disease management.

## MATERIALS AND METHODS

### Antimalarial compounds

For all studies reported in this manuscript, CBQ (free base, CAS 1469439-69-7) was supplied as a powder by Merck Healthcare KGaA, Darmstadt, Germany. Dihydroartemisinin (DHA) and atovaquone (ATQ) were purchased from APExBIO and Sigma, respectively. Stock solutions were prepared in DMSO at 10 mM. The final DMSO concentration used in all assays (<0.5%) had no inhibitory effect on parasites.

### Parasites

The following *P. falciparum* laboratory strains were used in this study: 3D7, NF54, a Dd2 line expressing the firefly *luciferase* reporter gene (Dd2^luc^) under the control of the *Pfhrp3* 5′UTR and *Pfhrp2* 3′UTR (35), and a Dd2 line expressing the NanoLuc reporter gene (Dd2^KnL^) under the control of the *Pfcalmodulin* 5′UTR and *Pfhrp2* 3′UTR (36).

### *P. falciparum* culture

Asexual blood-stage (ABS) parasites were cultured using standard methods as previously described. Briefly, these were cultured in A+ or O+ human red blood cells (RBCs) at 2%–3% hematocrit in RPMI-1640 medium supplemented with 25 mM HEPES, 0.5% AlbuMAX II, 0.21% sodium bicarbonate, 50 mg/L hypoxanthine, 2 mM L-glutamine, and 10 μg/mL gentamicin. Cultures were maintained at 37°C under a controlled gas atmosphere of 5% CO₂, 5% O₂, and 90% N₂.

### *Mitotracker in vitro* viability assessment assay

Asynchronous *P. falciparum* 3D7 cultures with a predominant ring population (ca. 70% rings) at 2% hematocrit and 0.5% parasitemia, distributed per well into 6-well plates, were incubated with the selected CBQ concentration (2.5 and 160 nM). Parasites were sampled at various time points: untreated (0 h) and treated parasites (2, 6, and 12 h). For each time point, the compound was removed by washing three times with incomplete medium (RPMI1640 supplemented with 25 mM HEPES, 1.77 mM sodium bicarbonate, and 12.5 μg/mL gentamicin sulfate) before preparing the samples for the direct viability assessment assay. As a control, an untreated sample was included to monitor parasite growth over a period of 12 h. Parasite viability at different time points post-drug incubation was quantified by flow cytometry after staining of parasites with SYBR Green (ThermoFisher Scientific) combined with MitoTracker Deep Red FM (ThermoFisher Scientific) as previously reported (17).

### Bioluminescence relative rate of kill assays

Two BRRoK reporter lines were used to assess CBQ’s effect on protein translation in rings and trophozoites, based on how closely their driving promoters overlap with the expression profile of PfeEF2, the molecular target of CBQ. PfeEF2 is broadly expressed starting in rings and peaking in trophozoites and early schizonts. As described below, the *Pfhrp3* promoter driving firefly luciferase overlaps more closely with the earlier part of this window, while the *Pfcalmodulin* promoter driving NanoLuc is more highly expressed later, providing an independent, complementary readout (PlasmoDB, version 68).

#### Firefly luciferase assay

A Dd2 parasite line expressing firefly *luciferase* (Dd2^luc^) under control of the *Pfhrp3* 5′UTR and *Pfhrp2* 3′UTR was used in this assay. The *Pfhrp3* 5′UTR is primarily active during ring and early trophozoite stages (peak 4–20 h post-invasion), as summarized from aggregate transcription data in PlasmoDB (version 68). Synchronized ring-stage (0–6 h post-invasion) or trophozoite-stage (24–32 h post-invasion) parasites were adjusted to 2% parasitemia and 2% hematocrit in 200 µL volumes.

Serial dilutions of CBQ, DHA, or ATQ (10 concentrations, 2-fold dilutions in duplicate) were prepared in pre-printed 96-well flat-bottom polystyrene plates (Fisher) using a Tecan D300e digital dispenser. The starting concentration of the compounds used in this study were as follows: CBQ (100 nM), dihydroartemisinin (DHA, 700 nM), atovaquone (ATQ, 500 nM). Drug-free (100% growth), and uninfected RBC (background bioluminescence) controls were included in each plate. Parasite cultures were then added manually to the plates and incubated for 6, 24, or 48 h at 37°C in a modular chamber incubator (Billups-Rothenberg) gassed with 5% O_2_, 5% CO_2_, and 90% N_2_.

For the 6 and 24 h timepoints, drug was removed by washing plates four times with complete media using a Tecan Freedom Evo 100 liquid handler. All plates were then further incubated until the 48 h endpoint. Plates were subsequently washed three times with 1 × PBS to remove residual compounds and RBC pellets were frozen at −80°C.

To measure bioluminescence, plates were thawed at 37°C for 10 min, and 100 µL of freshly prepared (by combining one vial of the lyophilized Bright-Glo Luciferase Assay Substrate with 10 mL Bright-Glo Luciferase Assay Buffer; Promega) Bright-Glo luciferase assay reagent was added to each well (18). Plates were incubated at room temperature for 2 min and transferred to 96-well white plates, and bioluminescence was measured on a SpectraMax M5 Multi-Mode Microplate Reader (Molecular Devices) with 1-s integration time. IC_50_ values were derived from growth inhibition data using linear interpolation and are reported as means ± SEM from three to six independent experiments, each performed in duplicate.

#### NanoLuc assay

A Dd2 parasite line expressing NanoLuc (Dd2^KnL^) under the control of the *Pfcalmodulin* 5′UTR and *Pfhrp2* 3′UTR was used. The *Pfcalmodulin* promoter, while active in rings, exhibits highest activity in late trophozoites and schizonts (32–40 h post-invasion), as summarized from aggregate transcription data in PlasmoDB (version 68). The assay setup, including parasite culture preparation, drug serial dilutions, incubation conditions, and drug removal, was identical to the firefly luciferase assay described above.

To measure bioluminescence, plates were thawed at 37°C for 10 min, and 100 µL of freshly prepared (by combining one vial of Nano-Glo Luciferase assay substrate [200 µL] with 10 mL Nano-Glo Luciferase assay buffer; Promega) Nano-Glo Luciferase assay reagent was added to each well. Plates were incubated at room temperature for 2 min and transferred to 96-well white plates, and bioluminescence was measured on a SpectraMax M5 Multi-Mode Microplate Reader (Molecular Devices) with 1-s integration time. IC_50_ values were calculated by linear interpolation as means ± SEM from two independent experiments, each performed in duplicate.

### Standard growth inhibition assays

#### SYBR Green and MitoTracker assay

Parasitemia was assessed in parallel assay plates at the 48 h endpoint using flow cytometry, using identical compound serial dilutions and parasite cultures to those described in the BRRoK assays. Plates were stained with 1 × SYBR Green and 100 nM MT Deep Red FM for 30 min at 37°C and then analyzed on an Intellicyt iQue Screener Plus (Sartorius). IC_50_ values were calculated by linear interpolation as means ± SEM from two independent experiments, each performed in duplicate.

#### Hypoxanthine incorporation assay

[^3^H]-hypoxanthine incorporation IC_50_ assays were performed according to the protocol published by Snyder et al. (37). Briefly, compounds were serially diluted in hypoxanthine-deficient medium in 96-well plates. iRBCs were added (final hematocrit of 1.25% and 0.3% parasitemia), and the assay plates were incubated for 48 h under cultivation conditions in humidified gas chambers. After 48 h, tritium- labeled hypoxanthine was added, and the assay plates were incubated for another 24 h. Radioactivity of incorporated tritium was measured using a BetaplateTM liquid scintillation counter. IC_50_ values were estimated using linear interpolation (37, 38).

Parasite growth after exposure to serial dilutions of antimalarial compounds was assessed using the [^3^H]hypoxanthine incorporation assay, and the level of inhibition of growth was expressed as the median (50%) inhibitory concentration (EC_50_) (32, 33). Exposures of 72 h were performed to allow the detection of slow-acting compounds and to enhance data reproducibility (39).

### Parasite culturing for imaging

Parasites were cultured at 5% CO_2_ and 3% O_2_ in a humidity-controlled environment and maintained at a hematocrit of about 2% in a 6 well dish; 50 µL blood and 2 mL complete RPMI (cRPMI) were added per well. cRPMI medium was prepared with 500 mL RPMI1640 (Thermo, 61870-010, GlutaMAX, Phenol, no HEPES), 10 mL 100× Hypoxanthine (CCPro Z-41-M 100 mL) [0.2 mM], 125 µL Gentamycin (Roth HN09.1 20 mL, 50 mgmL^−1^ [12.5 µgmL^−1^], 25 mL 10% Albumax (Life 11021-029, 25 g in 500 mL RPMI aliquots at −20°C) [0.5%], 5 mL HEPES 2.5 M stock, pH 7.3 [25 mM] (Roth 5.95g in 10 mL H_2_O). Parasitemia was maintained at ≤5% and monitored via Giemsa stain.

### Parasite seeding and drug treatment for imaging

Parasite cultures with a high parasitemia of about 5% (hematocrit of 2%) were used for seeding. For a surface treated quad dish (ibidi, 80416), around 1 mL of culture was spun down for 30 s at 1,000 g. The pellet was then washed with 1 mL sterile PBS and spun down again, followed by the addition of 1 mL sterile PBS. Two hundred microliters of this cell suspension was added to each well of the quad dish and incubated for 10 min. The wells were then gently washed with 200 µL of sterile PBS until monolayer remained on the bottom of each well. Five hundred microliters of cRPMI with 20 nM 5-SiR-Hoechst was added to the monolayer and incubated at 37°C for 3 h allowing the cells to take up the dye efficiently. Thereafter, the drug was added to the imaging wells in 100 µL cRPMI to 5 nM CBQ (Biozol, MCE-HY-117684) final concentration. For controls, 100 µL prewarmed cRPMI was added instead. Immediately afterward, the dish was transferred to the microscope incubation chamber with the lid being slightly open to allow for gas exchange. For Fluo-CBQ, the compound was carefully added in 100 µL cRPMI to the imaging dish on the microscope after acquisition of the pre-treatment image to 100 nM final concentration for single time point images taken after 10 min and 50 nM final concentration for short-term timelapse movies.

### Time-lapse imaging

For long-term live cell imaging, a Zeiss LSM 900 Airyscan 2 microscope in sensitivity mode with a 63 × 1.40 NA oil objective was used. The incubation chamber was heated to 37°C and gassed with 3% CO_2_ and 5% O_2_. The illumination settings for transmission channel 1 used T-DMT detector with 640 nm laser at 0.02% power, and for infrared channel 2, we used the Airyscan detector with 640 nm laser at 0.2% power at a scan speed 7 resulting in a pixel dwell time of 2.32 µs. Image settings were 56 nm pixel size, 454 × 454 pixels per image. The z-stacks were 6 µm with 13 slices at an interval of 500 nm. Multiply-infected RBCs were excluded from analysis. Images were acquired every hour for 60 h total. The image data were enhanced by Airyscan processing. The data were pooled from three independent replicates for each condition. For Fluo-CBQ treatment, all positions were acquired once prior to the addition of the compound. After addition of Fluo-CBQ, acquisition was immediately started with about 3.5 min time intervals acquired for up to 15 min. Measurements were carried out in four technical replicates and two biological replicates.

### Image analysis

Movies were manually annotated after drift correction and contrast adjustment using Fiji/Image J (version 1.54j). iRBCs without any clear hemozoin pigment were classified as ring stages. Small hemozoin-containing parasites were classified as early trophozoites and larger parasites with prominent hemozoin as late trophozoites. Schizonts were classified by checking whether more than one distinct nucleus could be detected. Nuclear multiplication was scored when multiple clearly distinguishable nuclei appeared. Egress was scored when iRBCs burst after the formation of distinct merozoites. This could be differentiated from RBC lysis, which occurs before parasites have reached the late schizont stage. A Hoechst spike was scored when the maximal fluorescence intensity within a parasite nucleus increased by more than fourfold within one- or two-time frames. For Fluo-CBQ quantification, integrated fluorescence intensity was measured using consistent circular consistent circular regions of interest (ROIs) for all timepoints on average intensity projections. After background subtraction the intensity values each cell were normalized by setting the maximum value to 1 and adjusting the other ones accordingly.

### Statistical analysis

All data are expressed as mean ± SEM from a minimum of two independent biological replicates, unless otherwise stated. For the live imaging, statistical comparisons used unpaired two-tailed Student’s *t*-test. For the BRRoK and standard growth inhibition assays, statistical comparisons between IC_50_ values were performed using an unpaired two-tailed Student’s *t*-test with Welch’s correction (Welch’s *t*-test) across all three assays (firefly luciferase, NanoLuc, and standard growth inhibition), reported as the primary test given unequal variances between compound groups. Welch’s *t*-test was chosen over the standard (equal-variance) Student’s *t*-test because group variances differed substantially across several compound/timepoint combinations, and Welch’s does not assume equal variances. To account for multiple comparisons, Holm-Šídák correction was applied within each timepoint where CBQ was compared against both DHA and ATQ (family of two tests sharing the same CBQ reference group); timepoints with only one testable comparison are reported uncorrected. Correction was applied per timepoint rather than across each full table: for the two BRRoK assays, ring- and trophozoite-stage measurements reflect different windows of promoter activity and are, therefore, not directly comparable biological readouts (*Pfhrp3* for luciferase, active in rings and early trophozoites but minimally active in the latter half of the cycle; *Pfcalmodulin* for NanoLuc, higher activity in trophozoites and schizonts). For the standard growth inhibition assay, ring- and trophozoite-stage values instead reflect overall parasite viability at a fixed 48 h endpoint rather than a real-time, promoter-dependent readout of translational status and are likewise treated as distinct comparisons rather than pooled. For the NanoLuc and standard growth inhibition assays, which were performed with only two independent experiments, resulting *P*-values should be interpreted with appropriate caution given the limited degrees of freedom. Where an IC_50_ could be determined in only one of two independent experiments (denoted ND in Tables S2 and S3), or where an IC_50_ was a censored “>” value with no defined mean/SEM, a *t*-test could not be performed and no *P*-value was reported for that condition (denoted n.t., not testable). Statistical significance was set at *P* < 0.05 (GraphPad Prism 8.0, GraphPad Software, La Jolla, CA, USA).

## Data Availability

All data are provided in the manuscript. Materials can be provided upon reasonable request to the corresponding authors.

## SUPPLEMENTAL MATERIAL

Supplemental material aac.00802-26-s0001.pdf Fig. S1 to S3; Tables S1 to S4.

ASM does not own the copyrights to Supplemental Material that may be linked to, or accessed through, an article. The authors have granted ASM a non-exclusive, world-wide license to publish the Supplemental Material files. Please contact the corresponding author directly for reuse.
