# Supplementary material for: In vitro pharmacodynamics of the antimalarial drug cabamiquine
Source: Antimicrob Agents Chemother. 2026 Sep 9;70(10):e00802-26. doi: 10.1128/aac.00802-26 (PMC13689320; doi:10.1128/aac.00802-26)
Supplement: Supplementary file 1 [file aac.00802-26-s0001.pdf]

SUPPORTING INFORMATION

In vitro pharmacodynamics of the antimalarial drug cabamiquine

Claudia Demarta-Gatsi[1#], Julia S. Wallner[2#], Satish K. Dhingra[3,4#], Sunil K. Narwal[3,4#], Vandana Thathy[3,4#], Mohamed Maiga[5], Maike Bonsen[2], Emilie Mathis[6], Ana Rita Gomes[6], Matthias Jacobs[7], Matthias Rottmann[8,9], Laurent Dembele[5], David A. Fidock[3,4,10]*, Julien Guizetti,[2†*] Thomas Spangenberg[1*]

#equal contributors

[1]Global Health R&D of Merck Healthcare, Ares Trading S.A., (a subsidiary of Merck KGaA, Darmstadt, Germany), Route de Crassier 1, 1262 Eysins, Switzerland.

[2]Centre for Infectious Diseases, Heidelberg University Hospital, 69120 Heidelberg, Germany.

[3]Department of Microbiology & Immunology, Room 1502 HHSC, 701 W. 168th St., Columbia University Irving Medical Center, New York, NY 10032, USA.

[4]Center for Malaria Therapeutics and Antimicrobial Resistance, Division of Infectious Diseases, Department of Medicine, Columbia University Irving Medical Center, New York, NY 10032, USA.

[5]Université des Sciences, des Techniques et des Technologies de Bamako (USTTB), Malaria Research and Training Centre (MRTC), Faculty of Pharmacy, Bamako, Mali.

[6] LPHI, Université de Montpellier, CNRS, INSERM, 34095 Montpellier, France.

[7]Merck KGaA, Darmstadt, Germany.

[8]Department of Medical Parasitology and Infection Biology, Swiss Tropical and Public Health Institute, Allschwil, Switzerland.

[9]University of Basel, Basel, Switzerland.

[10]Division of Infectious Diseases, Department of Medicine, Columbia University Irving Medical Center, New York, NY 10032, USA.

[†]Current address: Physical Chemistry of Biosystems, Institute of Physical Chemistry, Heidelberg University, 69120 Heidelberg, Germany

*Corresponding authors: DAF (df2260@cumc.columbia.edu), JG (julien.guizetti@uni-heidelberg.de), TS (Thomas.spangenberg@merckgroup.com)

Table of contents

Supplemental Text

- Fluo-CBQ synthesis and characterization:
  - Synthesis
  - LogD Determination,
  - Solubility,
  - Fluorescence
  - Cytotoxicity

Supplementary Figures:

- Figure S1. Comparative dose-response profiles of cabamiquine (CBQ), dihydroartemisinin (DHA) and atovaquone (ATQ) measured using the firefly luciferase assay (left; $Dd2^{luc}$) NanoLuc assay (middle; $Dd2^{KnL}$), and standard drug assay (right) in synchronized ring-stage parasites 0-6h post-invasion.

- Figure S2. Comparative dose-response profiles of cabamiquine (CBQ), dihydroartemisinin (DHA) and atovaquone (ATQ) measured using the firefly luciferase assay (left; $Dd2^{luc}$) NanoLuc assay (middle; $Dd2^{KnL}$), and standard drug assay (right) in synchronized trophozoite-stage parasites(24-32 h post-invasion)

- Figure S3. Effect of cabamiquine on egress and nuclear multiplication.

Supplementary Tables:

- Table S1. Mean ± SEM IC_{50} values of cabamiquine (CBQ), dihydroartemisinin (DHA) and atovaquone (ATQ) against the firefly luciferase-expressing $Dd2^{luc}$ parasite line.

- Table S2. Mean ± SEM IC_{50} values of cabamiquine (CBQ), dihydroartemisinin (DHA) and atovaquone (ATQ) against the NanoLuc-expressing $Dd2^{KnL}$ parasite line.

- Table S3. Mean ± SEM IC_{50} values of cabamiquine (CBQ), dihydroartemisinin (DHA) and atovaquone (ATQ) against the $Dd2^{luc}$ line as measured by standard growth inhibition assays with SYBR Green and MitoTracker dual labelling at 48 h.

- Table S4. Growth inhibition of Fluo-CBQ in *P. falciparum* NF54 after 72 h incubation.

Supplementary <u>Materials and Methods</u>

Fluo-CBQ synthesis and characterization

Synthesis

Synthesis of 2-((3-((5,5-difluoro-5H-4l4,5l4-dipyrrolo[1,2-c:2',1'-f][1,3,2]diazaborinin-10-yl)amino)propyl)amino)-6-fluoro-N-(2-(pyrrolidin-1-yl)ethyl)quinoline-4-carboxamide (fluo-Caba)

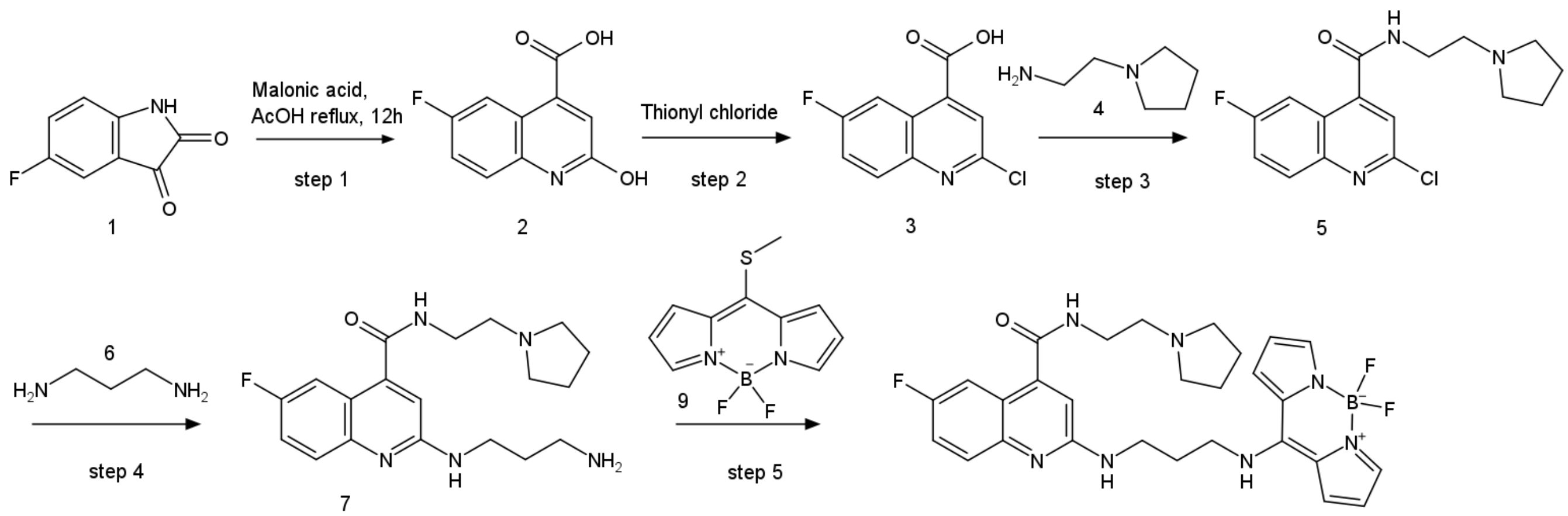

Step-1: A mixture of 5-fluoroindoline-2,3-dione **1** (2 g, 12.11 mmol) and malonic acid (3.78 g, 36.3 mmol) in acetic acid (60 mL) was stirred at 118 °C for 16 h. The progress of reaction was monitored by LCMS. After the completion of the reaction, the reaction was evaporated in vacuum to obtain solid. The solid was suspended in 10% Sodium bicarbonate solution (75 mL) and stirred for 15 mins. The formed suspension was filtered off and the MLR was acidified using conc. HCl solution to get a solid. The latter was isolated by filtration followed by drying to afford 6-fluoro-2-hydroxyquinoline-4-carboxylic acid **2** (2.11 g, 56.6%) as an off white solid. The solid used in the next step without further purification. ^{1}H NMR (400 MHz, DMSO) δ 8.06-8.02 (m, 1H), 7.58-7.37 (m, 2H), 7.01 (s, 1H), LC-MS (*m/z*) $[M + H]^+$ Calculated for $C_{10}H_6FNO_3$ 207.16, Observed 208.1.

Step-2: To a stirred solution of 6-fluoro-2-hydroxyquinoline-4-carboxylic acid **2** (2.1 g, 6.82 mmol) in DCM (60 mL), was added thionyl chloride (1.990 mL, 27.3 mmol) followed by DMF (1.056 mL, 13.64 mmol) at 0 °C. The reaction was stirred at 40 °C for 3 h. The reaction mixture was evaporated in vacuum to afford 2-chloro-6-fluoroquinoline-4-carbonyl chloride **3** (2.8 g, 78.0%) and taken as such for next step.

Step-3: To a stirred solution of 2-chloro-6-fluoroquinoline-4-carbonyl chloride **3** (1.66 g, 6.80 mmol) in THF (60 mL), was added 2-(pyrrolidin-1-yl)ethan-1-amine **4** (2.330 g, 20.41 mmol) at 0 °C. The reaction was stirred at room temperature for 16 h. The reaction mixture was evaporated in vacuum, diluted with 10% sodium bicarbonate solution (50 mL) and extracted in DCM (2 x 50 mL). The organic phase was dried over sodium sulphate and evaporated in vacuum to obtain 2-chloro-6-fluoro-*N*-(2-(pyrrolidin-1-yl)ethyl)quinoline-4-carboxamide **5** (2.05 g, 84 %) as an off white solid. ^{1}H NMR (400 MHz, DMSO) δ 8.87 (s, 1H), 8.12-8.03 (m, 2H), 7.84-7.79 (m, 1H), 7.69 (s, 1H), 3.49-3.45 (m, 2H), 2.67-2.64 (m, 2H), 2.55-2.50 (m, 4H), 1.75-1.72 (m, 4H), LC-MS (*m/z*) $[M + H]^+$ Calculated for $C_{16}H_{17}ClFN_3O$ 321.78, Observed 322.1.

Step-4: A mixture of 2-chloro-6-fluoro-*N*-(2-(pyrrolidin-1-yl)ethyl)quinoline-4-carboxamide **5** (250 mg, 0.777 mmol) and propane-1,3-diamine **6** (576 mg, 7.77 mmol) was stirred at 140 °C in MW for 1 h. The progress of reaction was monitored by LCMS. The reaction mixture was diluted with water (20 mL) and extracted in ethyl acetate (2 x 20 mL), dried over sodium sulphate and evaporated in vacuum to obtain 2-((3-aminopropyl)amino)-6-fluoro-*N*-(2-(pyrrolidin-1-yl)ethyl) quinoline-4-carboxamide **7** (240 mg, 78%) as an off white solid. ^{1}H NMR (400 MHz, DMSO) δ 8.87 (s, 1H), 8.12-8.03 (m, 2H), 7.84-7.79 (m, 1H), 7.69 (s, 1H), 3.49-3.45 (m, 2H), 2.67-2.64 (m, 2H), 2.55-2.50 (m, 4H), 1.75-1.72 (m, 4H), LC-MS (*m*/*z*) $[M + H]^+$ Calculated for $C_{19}H_{26}FN_5O$ 359.449, Observed 360.2 (M+1)

Step-5: To a stirred solution of 2-((3-aminopropyl)amino)-6-fluoro-*N*-(2-(pyrrolidin-1-yl)ethyl) quinoline-4-carboxamide **7** (0.240 g, 0.668 mmol) in ACN (5 mL), were added TEA (0.203 g, 2.003 mmol) and 5,5-difluoro-10-(methylthio)-5H-4l4,5l4-dipyrrolo[1,2-c:2',1'-f][1,3,2]diazaborinine (0.167 g, 0.701 mmol) **8** and the reaction was stirred at room temperature for 16 h. The reaction mixture was evaporated in vacuum to obtain crude. The crude was washed with ACN (25 mL), filtered off and dried in vacuum to obtain 2-((3-((5,5-difluoro-5H-4l4,5l4-dipyrrolo[1,2-c:2',1'-f][1,3,2] diazaborinin-10-yl)amino)propyl)amino)-6-fluoro-N-(2-(pyrrolidin-1-yl)ethyl)quinoline-4-carboxamide (**Fluo-CBQ)** (195 mg, 52.2 %) as an off white solid. ^{1}H NMR (400 MHz, DMSO) δ 7.65-7.59 (m, 2H), 7.53 (s, 1H), 7.37-7.31 (m, 4H), 6.88 (s, 1H), 6.38 (s, 2H), 3.97-3.93 (m, 2H), 3.74-3.70 (m, 2H), 3.62-3.59 (m, 2H), 2.80-2.76 (m, 2H), 2.69-2.66 (m, 4H), 2.29-2.26 (m, 2H), 1.88-1.85 (m, 4H), LC-MS (*m*/*z*) $[M + H]^+$ Calculated for $C_{28}H_{31}BF_3N_7O$ 549.409, Observed 550.3.

LogD Determination

Octanol saturated Phosphate Buffer 20 mM pH 7.4 was prepared by placing 100 mL Sorensen Phosphate Buffer 0.2 M pH 7.4 (EMS, Cat.-No. 11601-40) in a 1000 ml volumetric flask and ultrapure water was added to volume. Then 20 mL Octanol (Merck, Cat.-No.: 100991) to the Buffer was added, stirred overnight, and the phases were separated. Buffer saturated Octanol was prepared by adding 20 mL buffer (EMD, Cat.-No. 11601-40, as described above) to 1 L of Octanol (Merck, Cat.-No.: 1.00991), stirred overnight and the phases were separated.

Eight µL of 10 mM compound solution in DMSO was pipetted into a 0.5 mL safe-lock tube. Then 200 µL of the saturated Octanol and 200 µL of the saturated buffer solution were added. Overhead rotation was performed for 1 hr at RT. The sample was centrifuged for 2 min at 1000 rpm. 80 µL of the Octanol-phase (upper phase) was pipetted into an HPLC Vial and diluted with 720 µL methanol (dilution factor = 10). The buffer-phase (lower phase) will be taken with an insulin jab through the bottom of the safe-lock tube and injected in a HPLC (XBridge C8 3.5µm, 30 °C, UV 220, 254 and 290 nm, DAD detector). A suitable wavelength for evaluation of the chromatogram was selected to obtain maximum sensitivity to allow determination of high log D values (e.g. log D > 3.0). log D = log10 [(area Octanol * dilution factor Octanol / Injection volume Octanol) / (area buffer / Injection volume buffer)].

$$\text{Fluo-CBQ LogD}_{7,4} = 2.8$$

Solubility measurement

2.0 mg of compound was accurately weighed into a Uniprep® syringeless filter (5 mL 0.45 µm), and 2 mL of solvent was added and shaken for 24 h at 37°C. The pH was checked after 6 to 8 hours and if the pH deviated by more than 0.05 units, it was adjusted with 0.1 N HCl or 0.1 N NaOH solutions.

The suspension was filtered after 24 h and the concentration of dissolved substance was determined by HPLC after suitable dilutions with corresponding buffer if required. The result was expressed in µg/mL.

The solutions for these measurements were saturated solutions of respective compounds based on DMSO stock solutions (10 mM) in 20 mM PBS buffer pH 7.4 (same conditions as applied for K-Sol assay), with solid material of Fluo-CBQ that precipitated during the K-Sol assay being filtered off (0.2 µm filters) prior to fluorescence spectroscopy scans.

The DMSO co-solvent contribution from the K-Sol assay was reflected also in the respective blank measurements of the solvent.

Fluo-CBQ kinetic solubility = 81 µM (PBS pH 7.4, 2 h)

Cytotoxic assay

Fluo-CBQ was tested for in vitro cytotoxicity against a HepG2 cell-line by measuring depletion of ATP content (general marker of cytotoxicity) using a Promega® Celltiter Glo Assay. Compounds were tested in technical triplicate and samples were prepared from a 10 mM stock solution in DMSO. Metformin was used as negative control while tamoxifen was used as positive control. The 50% cytotoxic concentration (CC_{50}) values were obtained from dose-response curves.

Fluo-CBQ CC_{50} = 28 µM

Fluorescence Recording

The samples were measured using a Cary Eclipse fluorescence spectrometer by Agilent. Multiple emission spectra with increasing excitation wavelengths of each sample were recorded. For the measurements a standard 10x10mm quartz glass cuvette was used. The full parameters are contained in the table below.

In between each measurement the cuvette was rinsed with pure ethanol and dried using nitrogen. The detector's amplifier voltage was adjusted to 540V for Fluo-CBQ and 525V for the PBS Buffer so that the resulting spectra could be resolved without overloading the detector.

Parameter	Value
Wavelength Excitation [nm]	220-400
Wavelength Emission [nm]	220-700
Slit Excitation [nm]	5
Slit Emission [nm]	10
Step size Excitation [nm]	5
Step size Emission [nm]	4
Integration time [s]	0.4

Supplementary Figures

Figure S1. Comparative dose-response profiles of cabamiquine (CBQ), dihydroartemisinin (DHA), and atovaquone (ATQ) in rings. Synchronized ring-stage parasites (0-6 h post-invasion) were exposed to compounds for 6, 24, or 48 h. For the 6 and 24 h timepoints, compounds were washed off and plates incubated until the 48-h endpoint. Growth inhibition was assessed using three assays. **Left column:** Firefly luciferase activity ($Dd2^{luc}$) after 6 h **(A)**, 24 h **(D)**, or 48 h **(G)** compound exposure. **Middle column:** NanoLuc activity ($Dd2^{KnL}$) after 6 h **(B)**, 24 h **(E)**, or 48 h **(H)** compound exposure. **Right column:** Standard 48 h growth inhibition assays assessed by flow cytometry (SYBR Green and MitoTracker dual labelling) using the $Dd2^{luc}$ line after 6 h **(C)**, 24 h **(F)**, or 48 h **(I)** compound exposure. Data represent means ± SEM from 2-4 independent assays (luciferase and NanoLuc) or 2 independent assays (growth inhibition), each performed in duplicate. Dotted lines indicate 50% and 90% inhibition thresholds.

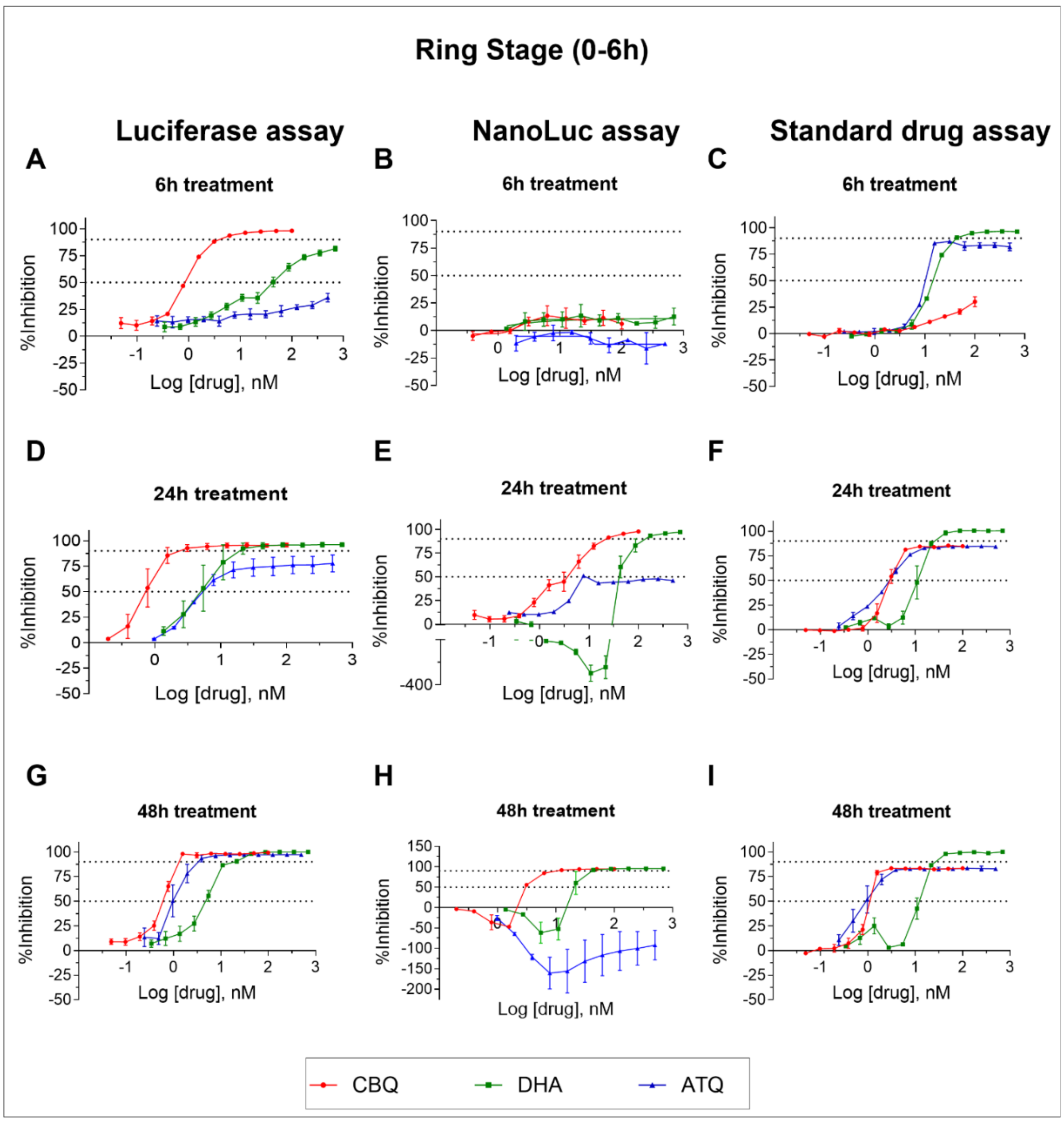

Figure S2. Comparative dose-response profiles of cabamiquine (CBQ), dihydroartemisinin (DHA) and atovaquone (ATQ) in trophozoites. Synchronized trophozoite-stage parasites (24-32 h post-invasion) were exposed to compounds for 6, 24, or 48 h. For the 6 and 24 h timepoints, compounds were washed off and plates incubated until the 48-h endpoint. Growth inhibition was assessed using three assays. **Left column:** Firefly luciferase activity ($Dd2^{luc}$) after 6 h **(A)**, 24 h **(D)**, or 48 h **(G)** compound exposure. **Middle column:** NanoLuc activity ($Dd2^{KnL}$) after 6 h **(B)**, 24 h **(E)**, or 48 h **(H)** compound exposure. **Right column:** Standard 48 h growth inhibition assays assessed by flow cytometry (SYBR Green and MitoTracker dual labelling) using the $Dd2^{luc}$ line after 6 h **(C)**, 24 h **(F)**, or 48 h **(I)** compound exposure. Data represent means ± SEM from 2-6 independent assays (luciferase and NanoLuc) or 2 independent assays (growth inhibition), each performed in duplicate.

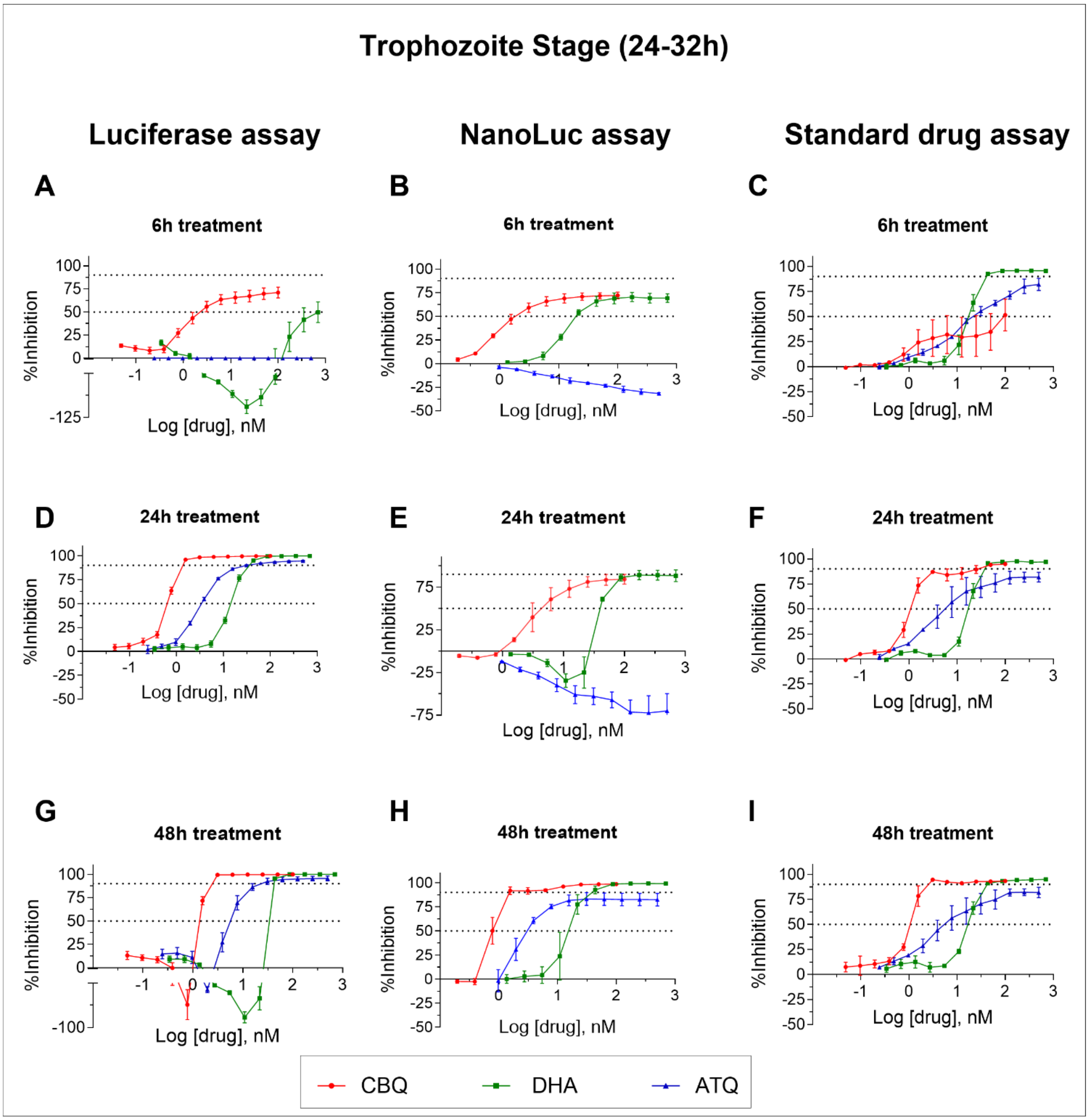

Figure S3. Effect of cabamiquine on egress and nuclear multiplication. A Percentage of all 5 nM cbamiquine-treated and control ring and trophozoite stages parasites showing nuclear multiplication or egress within the 60-h imaging time course. Data were pooled from three independent replicas for each condition.

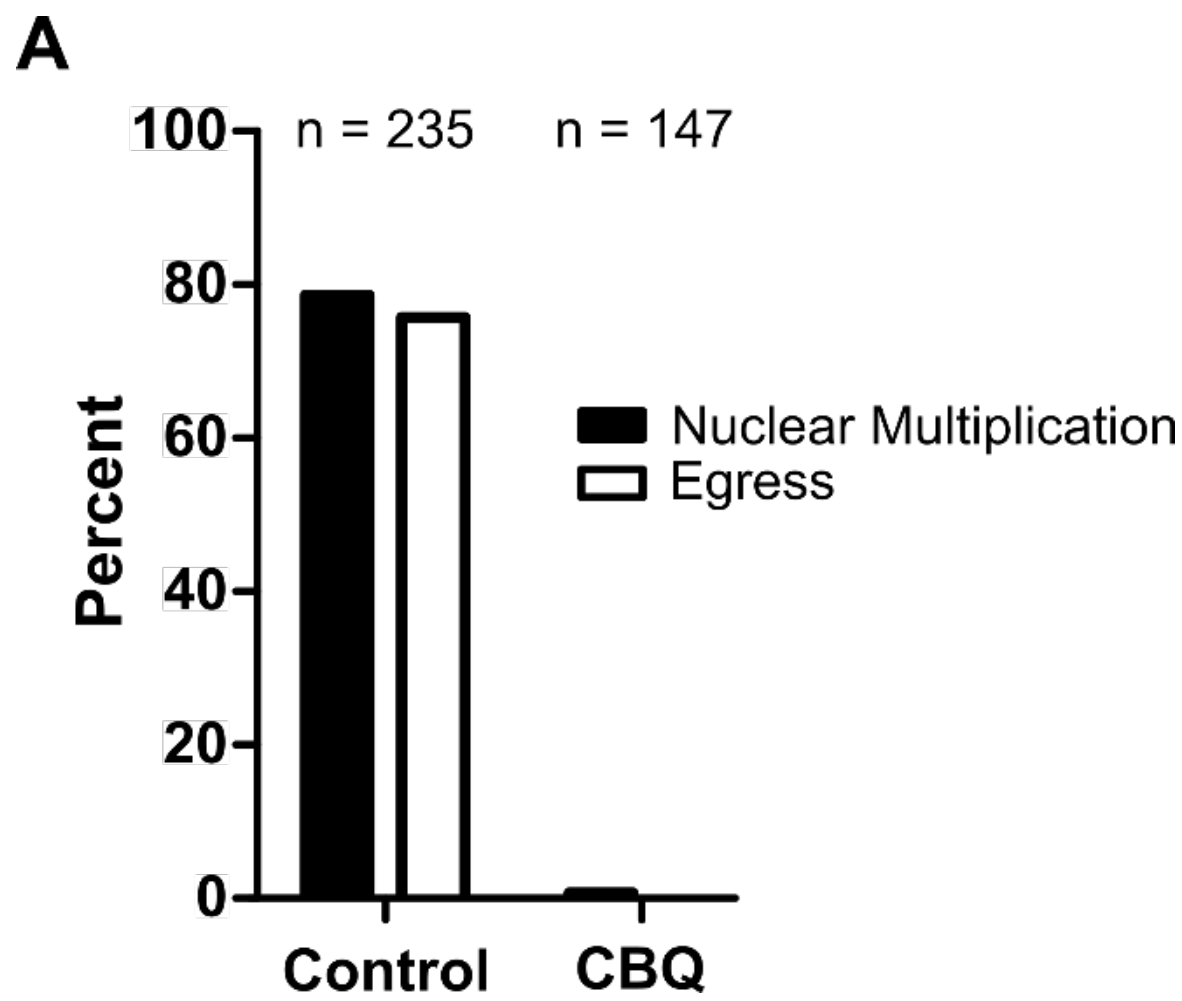

Supplementary Tables

Table S1. Mean ± SEM IC_{50} values of cabamiquine (CBQ), dihydroartemisinin (DHA) and atovaquone (ATQ) against the firefly luciferase-expressing $Dd2^{luc}$ parasite line.

Parasite stage	Treatment		CBQ	DHA	ATQ	*P* (CBQ vs DHA)	*P* (CBQ vs ATQ)
Rings (0-6 hpi)	6 h	N	3	4	4	0.077	n.t.
		IC_{50} (nM)	0.9 ± 0.1	46.8 ± 17.3	>500		
	24 h	N	3	4	3	0.104	n.t.
		IC_{50} (nM)	3.4 ± 2.3	54.0 ± 22.0	7.7 ± ND		
	48 h	N	3	3	4	0.114	0.529
		IC_{50} (nM)	0.6 ± 0.1	4.9 ± 1.1	1.1 ± 0.7		
Trophozoites (24-32 hpi)	6 h	N	4	5	5	0.223	n.t.
		IC_{50} (nM)	2.9 ± 1.4	166.2 ± 113.3	>500		
	24 h	N	5	6	6	0.0010*	0.0010*
		IC_{50} (nM)	0.7 ± 0.1	15.3 ± 1.9	3.6 ± 0.4		
	48 h	N	3	4	3	0.0070*	0.0948
		IC_{50} (nM)	1.4 ± 0.1	35.0 ± 4.0	5.9 ± 1.5		

*N, number of independent replicates. ND, not determined (IC_{50} could only be determined from 1 out of 3 independent experiments). Statistical significance (P) was determined using Welch's t-test with Holm-Šídák correction applied within each timepoint where CBQ was compared against both DHA and ATQ. n.t., not testable (one group's IC_{50} was a censored ">" value or had an undetermined SEM). *, $P < 0.05$ after Holm-Šídák correction within timepoint. Apparent differences in IC_{50} values between treatment durations, observed for CBQ, DHA, and ATQ, most likely reflect the fixed 48 h bioluminescence readout combined with variable drug-free recovery windows following washout, rather than genuine differences in compound potency. This effect is expected to be most pronounced for DHA given its short half-life under aqueous culture conditions (~2–6 h at 37°C; Parapini et al., 2015, Antimicrob Agents Chemother 59:4046–4052), such that nominal exposure duration in this assay reflects recovery time before readout more than cumulative active-drug exposure. The BRRoK assay measures reporter activity as a surrogate for translation inhibition rather than parasite viability, and IC_{50} values can additionally be influenced by reporter turnover, promoter activity, and dose-response curve fitting.*

Table S2. Mean ± SEM IC_{50} values of cabamiquine (CBQ), dihydroartemisinin (DHA) and atovaquone (ATQ) against the NanoLuc-expressing $Dd2^{KnL}$ parasite line.

Parasite stage	Treatment		CBQ	DHA	ATQ	*P* (CBQ vs DHA)	*P* (CBQ vs ATQ)
Rings (0-6 hpi)	6 h	N	2	2	2	n.t.	n.t.
		IC_{50} (nM)	>100	>700	>500		
	24 h	N	2	2	2	0.299	0.093
		IC_{50} (nM)	0.8 ± 0.2	6.1 ± 2.7	5.9 ± 0.6		
	48 h	N	2	2	2	0.163	n.t.
		IC_{50} (nM)	3.0 ± 0.01	23.6 ± 5.4	>500		
Trophozoites (24-32 hpi)	6 h	N	2	2	2	0.0085*	n.t.
		IC_{50} (nM)	2.0 ± 0.5	20.4 ± 0.9	>500		
	24 h	N	2	2	2	0.0407*	n.t.
		IC_{50} (nM)	5.1 ± 2.3	41.0 ± 0.04	>500		
	48 h	N	2	2	2	0.195	0.195
		IC_{50} (nM)	0.8 ± 0.1	15.2 ± 3.8	3.0 ± 0.4		

*N, number of independent replicates. Statistical significance (P) was determined using Welch's t-test with Holm-Šídák correction applied within each timepoint where CBQ was compared against both DHA and ATQ. n.t., not testable (one group's IC_{50} was a censored “>” value). *, $P < 0.05$ after Holm-Šídák correction within timepoint. Apparent differences in IC_{50} values between treatment durations, observed for CBQ, DHA, and ATQ, most likely reflect the fixed 48 h bioluminescence readout combined with variable drug-free recovery windows following washout, rather than genuine differences in compound potency. This effect is expected to be most pronounced for DHA given its short half-life under aqueous culture conditions (~2–6 h at 37°C; Parapini et al., 2015, Antimicrob Agents Chemother 59:4046–4052), such that nominal exposure duration in this assay reflects recovery time before readout more than cumulative active-drug exposure. The BRRoK assay measures reporter activity as a surrogate for translation inhibition rather than parasite viability, and IC_{50} values can additionally be influenced by reporter turnover, promoter activity, and dose-response curve fitting.*

Table S3. Mean ± SEM IC_{50} values of cabamiquine (CBQ), dihydroartemisinin (DHA) and atovaquone (ATQ) against the $Dd2^{luc}$ line as measured by standard growth inhibition assays with SYBR Green and MitoTracker dual labelling at 48 h.

Parasite stage	Treatment		CBQ	DHA	ATQ	*P* (CBQ vs DHA)	*P* (CBQ vs ATQ)
Rings (0-6 h)	6 h	N	2	2	2	n.t.	n.t.
		IC_{50} (nM)	>100	15.4 ± 0.1	10.9 ± 0.1		
	24 h	N	2	2	2	0.446	1.000
		IC_{50} (nM)	3.0 ± 0.5	12.0 ± 3.9	3.0 ± 0.2		
	48 h	N	2	2	2	0.292	0.758
		IC_{50} (nM)	1.2 ± 0.03	12.6 ± 2.9	1.0 ± 0.5		
Trophozoites (24-32 h)	6 h	N	2	2	2	n.t.	n.t.
		IC_{50} (nM)	17.6 ± ND	18.2 ± 3.2	23.8 ± 8.0		
	24 h	N	2	2	2	0.133	0.482
		IC_{50} (nM)	1.2 ± 0.2	18.1 ± 1.9	8.4 ± 6.8		
	48 h	N	2	2	2	0.124	0.504
		IC_{50} (nM)	1.1 ± 0.1	17.8 ± 1.7	9.4 ± 8.4		

N, number of independent replicates. ND, not determined (IC_{50} could only be determined from 1 out of 2 independent experiments). Statistical significance (P) was determined using Welch's t-test with Holm-Šídák correction applied within each timepoint where CBQ was compared against both DHA and ATQ. n.t., not testable (one group's IC_{50} was a censored “>” value or had an undetermined SEM). No significant differences were observed between groups after Holm-Šídák correction within timepoint.

Table S4. Growth inhibition of **Fluo-CBQ** in *P. falciparum* NF54 after 72 h incubation.

Compound ID	***IC_{50} (ng/ml; n=3)***
Fluo-CBQ	78
Chloroquine	**3.7**
Artesunate	**0.47**
